# CK2-NCoR signaling cascade promotes prostate tumorigenesis

**DOI:** 10.18632/oncotarget.1020

**Published:** 2013-05-11

**Authors:** Jung-Yoon Yoo, Beom Jin Lim, Hyo-Kyoung Choi, Soon Won Hong, Ho Sung Jang, Changsoo Kim, Kyung-Hee Chun, Kyung-Chul Choi, Ho-Geun Yoon

**Affiliations:** ^1^ Department of Biochemistry and Molecular Biology, Brain Korea 21 Project for Medical Sciences, Yonsei University College of Medicine, Seoul 120-752, Korea; ^2^ Department of Pathology, Yonsei University College of Medicine, Seoul 120-752, Korea; ^3^ Department of Urology, Urological Science Institute, Yonsei University College of Medicine, Seoul, Korea; ^4^ Department of Preventive Medicine, Yonsei University College of Medicine, Seoul, Korea; ^5^ Genitourinary Cancer Branch, Division of Translational and Clinical Research II, National Cancer Center Research Institute and Hospital, Ilsandong-gu, Goyang-si, Gyeonggi-do, Korea; ^6^ Asan Medical Center, University of Ulsan College of Medicine, 388-1 Poongnap-DONG, Songpa-gu, Seoul 138-736, Korea

**Keywords:** CK2α, NCoR, Prostate cancer, IP-10, transcriptional regulation

## Abstract

The aberrant expressions of casein kinase 2 (CK2) was found in prostate cancer patient and cell lines, but little is known of the detailed mechanisms implicated in prostate tumorigenesis. In this study, we report that both CK2 activity and CK2-mediated NCoR phosphorylation are significantly elevated in the androgen-independent prostate cancer cell line DU145 and PC-3 compared with RWPE1 and LNCaP cells. Increased phosphorylation inversely correlates with the mRNA level of the NCoR-regulated gene, interferon-γ-inducible protein 10 (IP-10). CK2 inhibition abrogated NCoR phosphorylation, IP-10 transcriptional repression, and the invasion activity of PC-3 cells. Inhibition of the CK2-NCoR network significantly reduced in vivo PC-3 cell tumorigenicity, likely due to transcriptional derepression of IP-10. Clinicopathological analyses revealed that increased CK2-mediated NCoR phosphorylation significantly correlates with poor survival among prostate cancer patients. These findings elucidate a CK2-modulated oncogenic cascade in prostate tumorigenesis.

## INTRODUCTION

Casein kinase 2 (CK2) is a multifunctional protein kinase with a wide range of protein substrates, many of which are critically involved in the processes of cell cycle control, cellular differentiation, proliferation, and metabolism [1]. Moreover, CK2 can exert anti-apoptotic effects through various mechanisms [2-4]. Thus, dysregulation of CK2 in tumor cells may influence apoptotic activity and enhance cell survival [5, 6]. Overexpression of CK2α leads to increased c-myc expression in T cell lymphoma [7], NFκB activation in NIH3T3 cells [8], and protects PC-3 cells from etoposide-induced apoptosis [9]. Overexpression of CK2β in 3T3-L1 fibroblasts results in an increased growth rate [10]. In transgenic mice, CK2α overexpression cooperates with c-myc or p53 loss (or mutation) at the lpr locus to promote tumorigenesis [11]. Transgenic expression of CK2α under the MMTV promoter resulted in late onset squamous adenocarcinomas with increased c-myc and β-catenin expression [12]. Moreover, CK2 participates in the control of Snail1, a major factor for epithelial-mesenchymal transition, by stabilizing and positively regulating Snail1 repressive function and its interaction with the mSin3A corepressor [13]. Therefore, CK2 is recently highlighted as promising target for cancer therapies [5, 14, 15].

Recent studies have demonstrated that CK2 expression and activity are highly elevated in multiple tumors and tumor cell lines [9, 16]. For example, two esophageal cancer cell lines, TE2 and HCE4, displayed opposing CK2 activities, but similar CK2 expression levels [17, 18]. This differential pattern of CK activity correlated with the invasive growth of esophageal cancer cells [18]. In addition, the androgen-independent prostate cancer cell line PC-3 also displays relatively elevated CK2 activity compared to LNCaP prostate cells [19, 20]. In accordance with these observations, targeting CK2α with antisense RNA or inhibitors induces tumor shrinkage in a human prostate cancer xenograft model, suggesting the importance of CK2 in prostate tumorigenesis [3, 21, 22]. Although the importance of CK2 in tumor cell survival and growth is clear, the molecular mechanism by which CK2 is involved in prostate tumorigenesis has not been extensively investigated.

Nuclear receptor corepressor (NCoR) forms corepressor complexes with histone deacetylase 3 (HDAC3) to induce changes in local chromatin structure and thereby cause transcriptional repression [23-25]. NCoR interacts with antagonist-bound androgen receptor (AR) to repress its activity, which is believed to be the basic principle for the current widespread use of hormone therapy for prostate cancer [26, 27]. During the progression to castration-resistant prostate cancer (CRPC), coactivators such as SRC-1 and TIF-2 greatly contribute to the growth of androgen-independent prostate cancer cells by reactivation of AR by coactivators which are highly overexpressed in recurrent prostate cancer compared with benign prostatic hyperplasia or androgen-dependent prostate cancer [28]. However, the role of AR corepressors during androgen-independent prostate cancer progression is still unclear. In this regard, we recently proposed an oncogenic cascade where both CK2 and NCoR selectively repress the transcription of a sub-set of target genes including the anti-tumorigenic gene interferon-γ-inducible protein 10 (IP-10), to promote oncogenic signaling in human cancer cells [18]. Although we suggested the molecular basis for CK2-NCoR cascade for tumorigenic growth of cancer cells *in vitro*, the clinical implications of this cascade in human cancer development is currently unknown.

In this study, we demonstrate the clinical relevance of the CK2-NCoR axis in prostate cancer development and provide a mechanism related to the invasive growth of malignant prostate cancer cell, PC-3. Furthermore, we show that blocking the CK2-NCoR network suppresses the *in vivo* tumorigenicity of prostate cancer cells via derepression of IP-10 mRNA, which may provide a potent therapeutic strategy for the treatment of prostate tumor progression.

## RESULTS

### CK2-mediated NCoR phosphorylation is elevated in PC-3 cells

**Figure 1:**
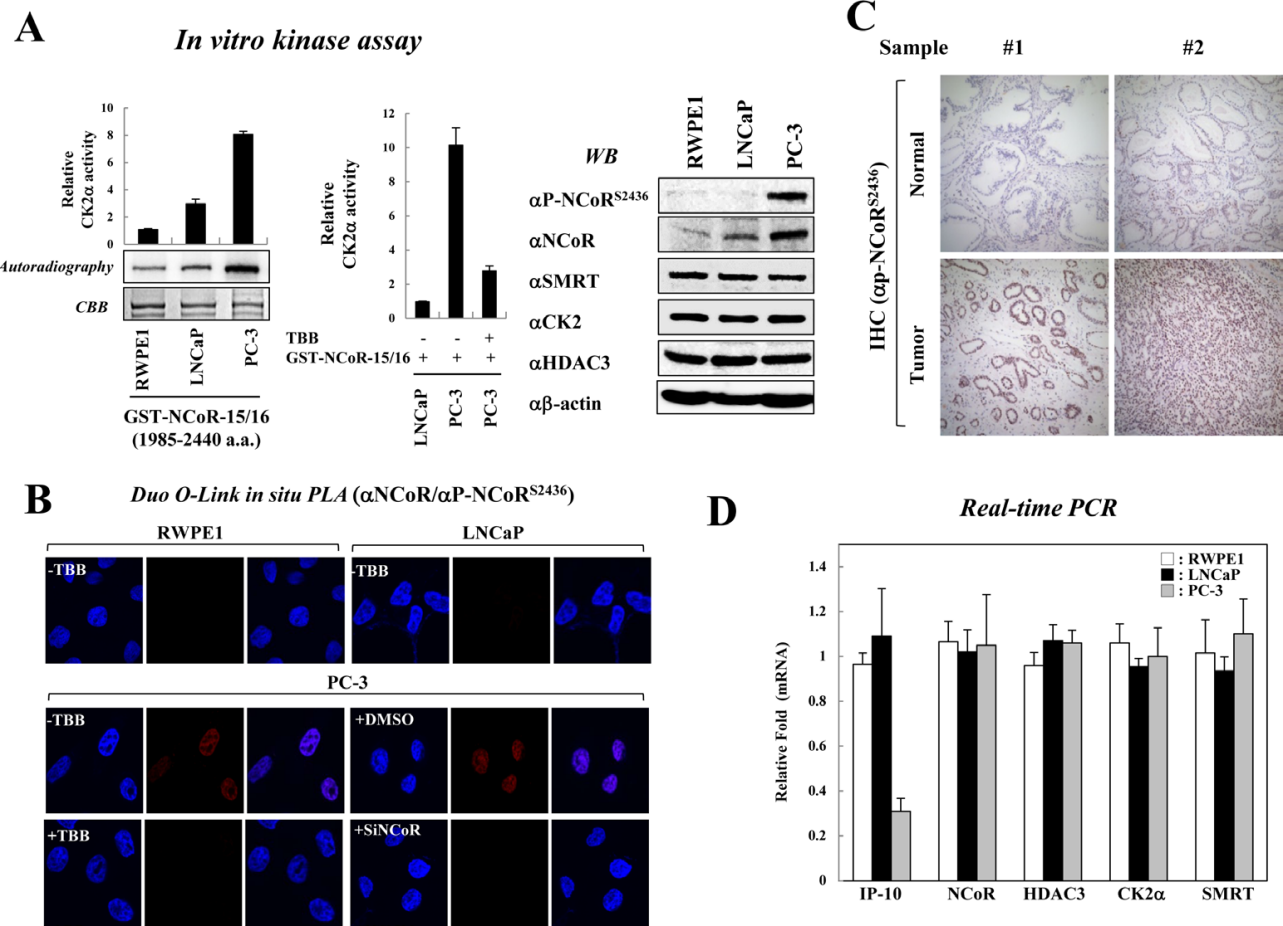
Constitutive activation of CK2 and NCoR^S2436^ phosphorylation in PC-3 cells (A) *In vitro* kinase assays were performed by incubating GST-NCoR-15/16 protein and immunoprecipitated CK2 enzyme obtained from indicated prostate cancer cells. NCoR phosphorylation levels were analyzed by autoradiography and scintillation counts (left & middle panel). Error bars indicate SD (n=3). Cell lysates from prostate cancer cells were analyzed by Western blotting (right panel). (B) For the Duolink in situ PLA analysis, indicated cells were seeded on coverslips and treated with the siNCoR in the presence of DMSO or TBB (50 μm). The pre-metabolized cells were incubated with the indicated antibodies and treated with PLA probes (PLUS and MINUS). The positive signal was analyzed using confocal microscopy. (C) Expression of NCoR^S2436^ phosphorylation was examined using immunohistochemical staining in samples from prostate cancer patients. Representative NCoR^S2436^ phosphorylation levels are shown in two normal and tumor sections. The nuclei were counterstained with hematoxylin. (D) Expression levels of indicated genes from prostate cancer cells were analyzed by real-time PCR. Error bars indicate SD (n=3).

Recent studies demonstrated that NCoR expression is frequently elevated in malignant prostate cells compared with non-malignant cells [29, 30]. Furthermore, aberrant expression of CK2 has been reported in prostate cancers [19, 20, 31]. Therefore, we first assessed the relative activity of CK2 in among various prostate cell lines, including a non-transformed prostate epithelial cell line (RWPE1), an androgen-dependent prostate cancer cell line (LNCaP), and an androgen-independent prostate cancer cell line (PC-3). In vitro kinase assays revealed that CK2 activities in PC-3 cells are significantly higher than in both RWPE1 and LNCaP cells (Fig. 1A, left panel); however, CK2α inhibitor TBB treatment of PC-3 cells decreased CK2 activity (Fig. 1A, middle panel). Next, we examined the relative level of CK2-mediated NCoR phosphorylation among these cell lines with a phospho-specific NCoR antibody that specifically recognizes phosphorylated Ser-2436 of NCoR as described previously [18]. Intriguingly, significant levels of both NCoR and phosphor-NCoR^S2436^ were observed in PC-3 cells compared with LNCaP and RWPE1 (Fig. 1A, right panel). This correlates with the results from an *in vitro* kinase assay, suggesting constitutive activation of the CK2-NCoR cascade in PC-3 cells (Fig. 1A). We also observed no significant difference in CK2 and HDAC3 levels among these cells (Fig. 1A). It is also noteworthy that the levels of NCoR and NCoR phosphorylation is elevated in LNCaP-derivative C4-2B cells compared with LNCaP cells, although to a less than both DU145 and PC-3 cells, implying the plausible role of CK2-NCoR cascade in hormone independency of prostate cancer cells (Supplementary Figure 1). Importantly, DuoLink *in situ* proximity ligation assay (PLA) analysis verified elevated levels of NCoR^S2436^ phosphorylation in PC-3 cells (Fig. 1B). Consistently, TBB efficiently blocked NCoR ^S2436^ phosphorylation in PC-3 cells, further confirming elevated CK2-NCoR signaling in PC-3 cells. To corroborate this finding, we have examined whether CK2-mediated NCoR phosphorylation is elevated in prostate cancer patients. As shown in Fig. 1C, immunohistochemical analyses demonstrate elevated levels of phosphor-NCoR^S2436^ in tumor regions compared with adjacent non-neoplastic prostate tissues, suggesting a possible role for the CK2-NCoR signaling cascade in prostate tumorigenesis.

The IP-10/CXCL10 gene was identified as a CK2 and NCoR network-regulated gene [18]. Therefore, we assessed the mRNA level of IP-10 in prostate cancer cells. Real-time PCR analyses demonstrated reduced levels of IP-10 mRNA in PC-3 cells compared with RWPE1 and LNCaP cells, which inversely correlates with CK2-mediated NCoR phosphorylation (Fig. 1D). This data supports the hypothesis that CK2 phosphorylates NCoR to repress the transcription of IP-10 in PC-3 cell. In addition, ONCOMINE database analysis indicated decreased mRNA levels of IP-10 in prostate cancer patients compared with normal patients or patients with other cancer types (Supplementary Figure 2). Together, these data suggest that the CK2-NCoR oncogenic cascade, at least in part, is involved in prostate cancer tumorigenesis via transcriptional repression of IP-10.

### The CK2-NCoR network promotes invasive growth of PC-3 Cells

**Figure 2:**
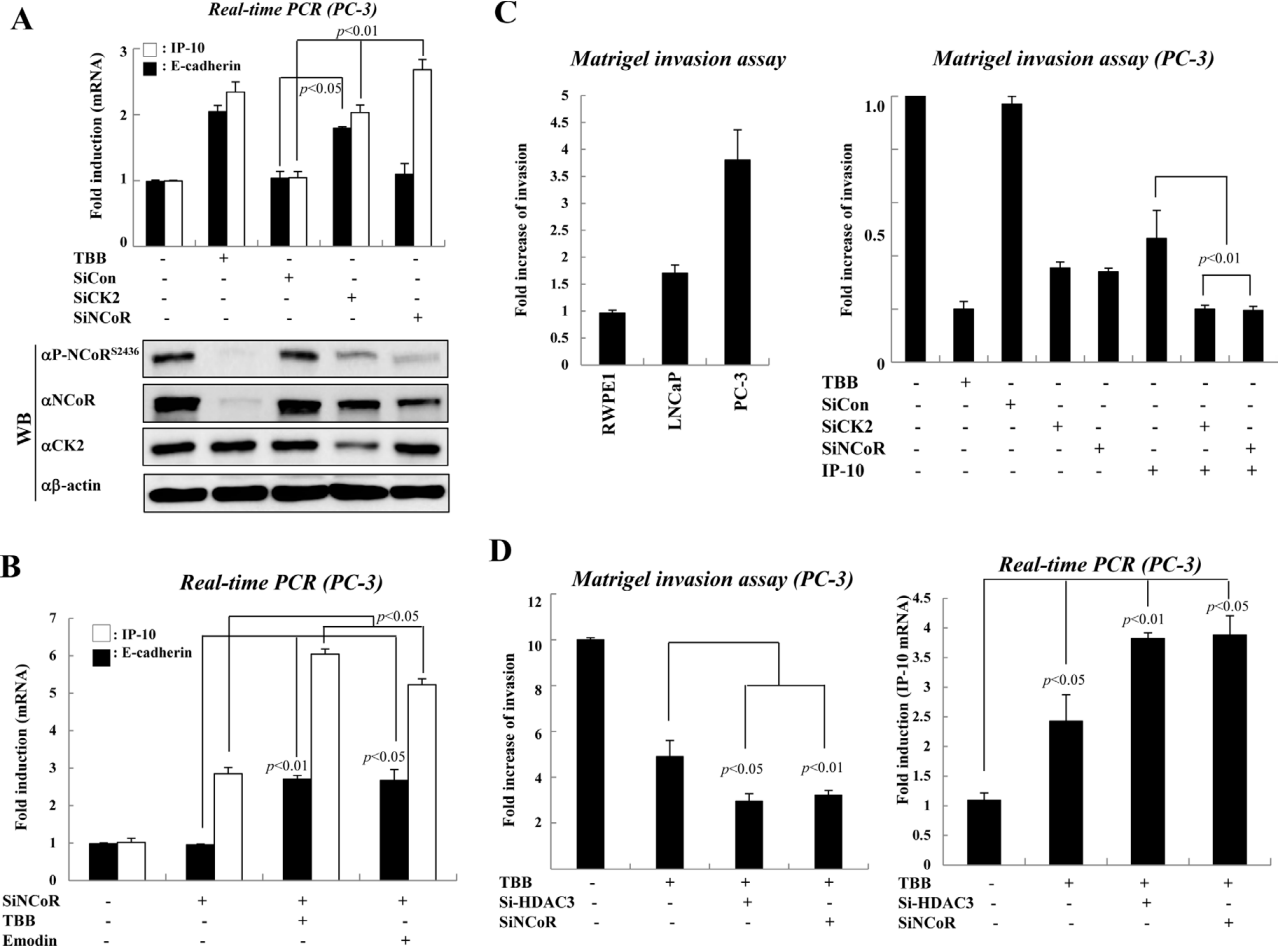
The activated CK2-NCoR network promotes invasion activity of PC-3 cells (A) PC-3 cells were treated with TBB (50 μM, 6 hr) or the indicated siRNAs and cDNA was prepared for real-time PCR. Error bars indicate SD (n=3). Cell lysates were analyzed by Western blotting. (B) PC-3 cells were treated with CK2 inhibitors (TBB, emodin) or transfected with siNCoR. cDNA was prepared for real-time PCR analysis. Error bars indicate SD (n=3). (C) Invasive growth of prostate cancer cells was analyzed by counting cells that migrated through the extracellular matrix layer of Biocoat Matrigel invasion chambers. Error bars indicate SD (n=3) (left panel). PC-3 cells were transfected with the indicated siRNAs and expression plasmids in the presence or absence of TBB before application to the upper chamber. Error bars indicate SD (n=3) (right panel). (D) PC-3 cells were transfected with the indicated siRNAs or treated with TBB. Invasive growth was analyzed by counting cells that migrated through the extracellular matrix layer of Biocoat Matrigel invasion chambers. Error bars indicate SD (n=3) (left panel). The level of IP-10 mRNA was determined by real-time PCR. Error bars indicate SD (n=3). (right panel).

To elucidate the relationship between CK2-NCoR signaling and prostate tumorigenesis, we first investigated whether the depletion of NCoR selectively represses IP-10 transcription in PC-3 cells. As shown in Fig. 2A, inhibition or depletion of CK2 derepressed the transcription of both E-cadherin and IP-10. However, NCoR knockdown selectively derepressed IP-10, confirming that the CK2-NCoR network selectively represses IP-10 transcription in PC-3 cells. It is noteworthy that TBB treatment dramatically reduced the elevated levels of both NCoR and phosphor-NCoR^S2436^ in PC-3 cell (Fig. 2A), again emphasizing the role of CK2 on NCoR stabilization. In addition, treating cells with another CK2α inhibitor, Emodin, in combination with siNCoR, also verified the selective repression of IP-10 transcription by the CK2-NCoR network (Fig. 2B).

Consistent with increased CK2 activity and NCoR phosphorylation, we also observed increased invasion activity in PC-3 cells compared with RWPE1 and LNCaP cells (Fig. 2C, left panel). This increased invasion activity of PC-3 cells seemed to be dependent on CK2 activity, since either siCK2 or TBB treatment significantly impaired the invasion activity of PC-3 cells (Fig. 2C). Importantly, combinatorial treatment of siCK2 or siNCoR with overexpression of IP-10 further suppressed the invasive growth of PC-3 cells, suggesting the functional significance of CK2-NCoR-IP-10 signaling in tumorigenic growth of PC-3 cells (Fig. 2C, right panel and Supplementary Figure 3A).

Since the repressive function of the NCoR corepressor complex depends on HDAC3[25], we next assessed the effect of knocking down HDAC3 on the invasive growth of PC-3 cells. As shown in Fig. 2D (left panel), depletion of NCoR synergistically suppressed the invasive growth of PC-3 cells when treated with TBB. Combinatorial treatment of TBB with siHDAC3 also displayed a synergistic effect on invasive growth of PC-3 cells, indicating a functional engagement of the NCoR-HDAC3 corepressor complex in invasive growth of PC-3 cells (Supplementary Figure 3B). As expected, we also observed enhanced derepression of IP-10 upon combinatorial treatment of TBB and siHDAC3 (Fig. 2D, right panel).

### Decreased expression of IP-10 in PC-3 cells is associated with enhanced recruitment of the NCoR-HDAC3 corepressor complex to the AP-1 site of IP-10 gene

**Figure 3:**
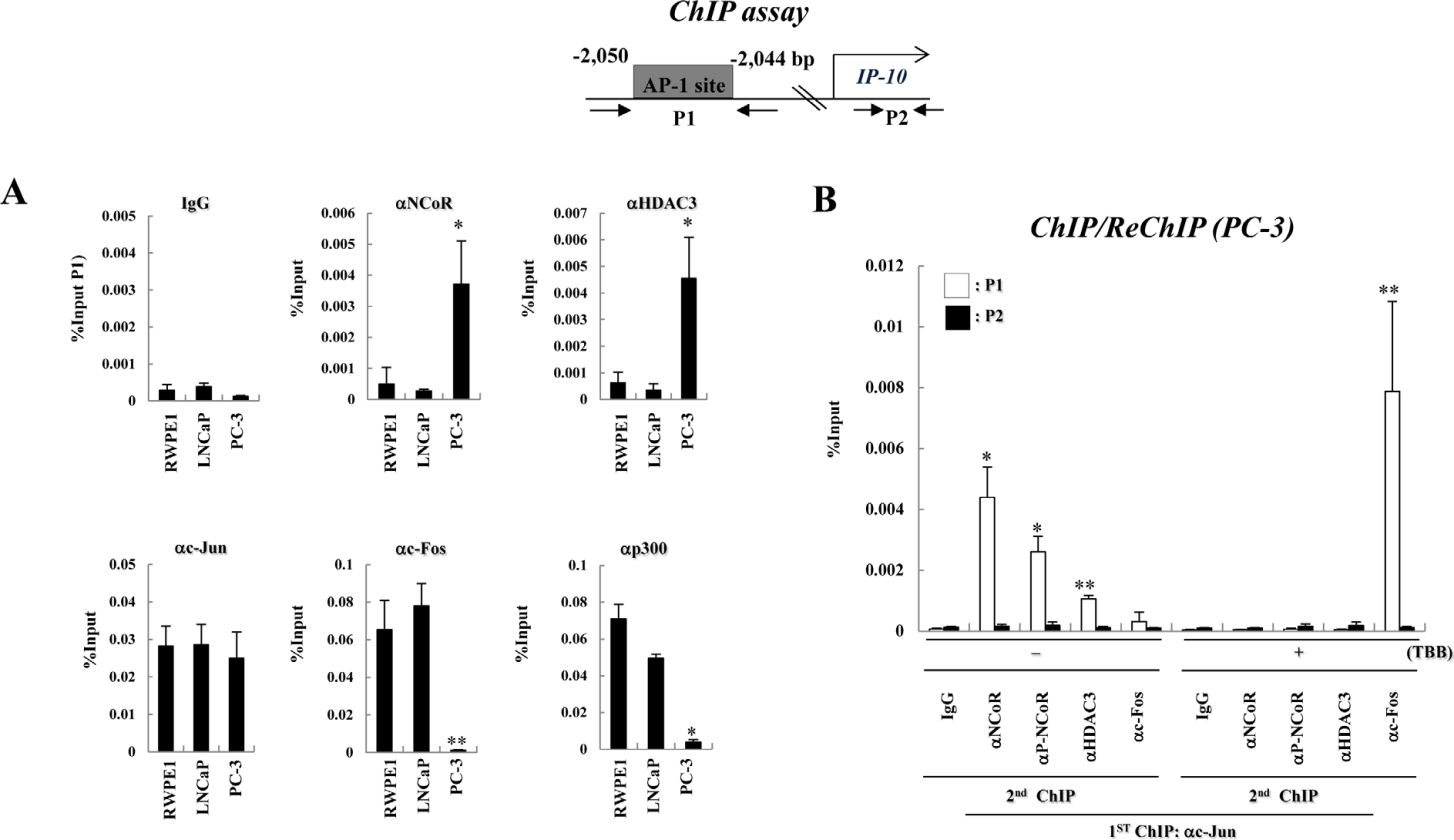
CK2 activity is required for the recruitment of the NCoR-HDAC3 corepressor complex to AP-1 site of the IP-10 promoter in PC-3 cells (A) A diagram of the IP-10 promoter showing the position of the AP-1 site and the primers used for real-time PCR analyses in ChIP assays. ChIP assays were performed with the indicated antibodies. The precipitated samples were analyzed by real-time PCR, and results are given as the percentage of input (mean ± SD of three independent experiments). Error bars indicate SD of three independent sets. * p<0.01 vs. LNCaP; ** p<0.001 vs. LNCaP (B) PC-3 cells were treated with or without TBB and ChIP/reChIP assays were performed with the indicated antibodies. Error bars indicate SD of three independent sets. * p<0.05; ** p<0.01.

Because we observed constitutively elevated levels of CK2 activity in PC-3 cells and a corresponding decrease in IP-10 transcription, we investigated the relative occupancy of the NCoR-HDAC3 corepressor complex in the IP-10 gene promoter region in prostate cancer cells. We previously identified a c-Jun-binding AP-1 site in the IP-10 gene promoter at position -2,044 to -2,050 (relative to the transcription start site) [18]. Chromatin immunoprecipitation (ChIP) experiments indicated the recruitment of NCoR and HDAC3 to the IP-10 promoter was increased in PC-3 cells compared with RWPE1 and LNCaP cells (Fig. 3A). In contrast, increased occupancy of the c-Fos and p300 promoters was observed in RWPE1 and LNCaP cells. These data suggest differential recruitment of the NCoR-HDAC3 corepressor complex to the AP-1 site of the IP-10 gene promoter determines the level of IP-10 mRNA, which correlates with invasion activity of prostate cancer cells.

Next, we examined whether CK2 activity is required for increased NCoR-HDAC3 corepressor complex binding to the AP-1 site of the IP-10 promoter. As shown in Fig. 3B, ChIP and ReChIP analyses demonstrated that both NCoR and HDAC3 associate with c-Jun in the AP-1 site to repress IP-10 transcription, whereas c-Fos is dissociated from c-Jun. Importantly, TBB treatment induced NCoR corepressor complex dissociation from the AP-1 site as well as increased formation of the c-Jun/c-Fos activating complex. These data suggest that enhanced CK2 activity in PC-3 cells stabilizes the NCoR protein and subsequently increases the formation of c-Jun-NCoR-HDAC3 corepressor complex in the AP-1 site to constitutively repress IP-10 transcription.

### CK2-NCoR pathway inhibition suppresses in vivo tumorigenesis of PC-3 cells via repression of IP-10

**Figure 4:**
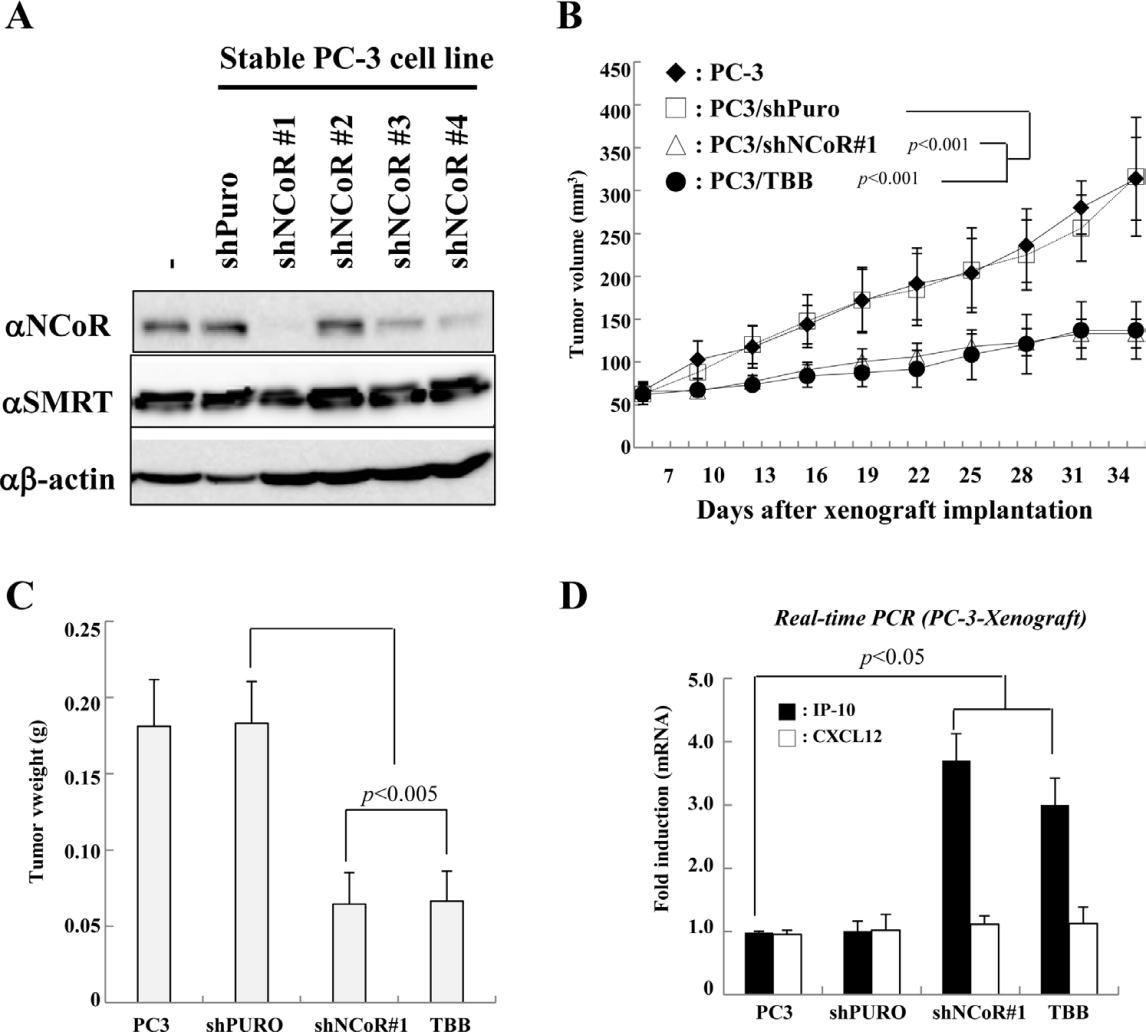
Effect of CK2-NCoR axis inhibition on *in vivo* tumorigenecity of PC-3 cells (A) Stable PC-3 cells that express shNCoR were generated as described in the Materials and Methods. The cell lysates were subsequently immunoblotted with the indicated antibodies. (B-C) Stably transfected PC-3 cells (1.5 × 10^6^ cells) were injected subcutaneously into the right flank of nude mice. Mice were sacrificed 9 weeks post injection. Tumor volume (B) and weight (C) were measured for 9 weeks. Values give are the mean ±SD for eight mice from a representative experiment. (D) Mice were sacrificed and tumor tissues were collected, processed, and subjected to real-time PCR. Error bars indicate SD (n=3).

Given the critical role of CK2-NCoR signaling in the *in vitro* tumorigenicity of PC-3 cells, we next examined whether inhibition of CK2 and NCoR inhibits their *in vivo* tumorigenecity. To this end, we generated several stable PC-3 cell lines with NCoR knocked down using lentiviral shRNAs and selected the cell line with the most efficient lentiviral shRNA against NCoR (Fig. 4A). As shown in Fig. 4B, a xenograft assay using subcutaneous injection of PC-3 cells into nude mice demonstrated that TBB treatment or stable knockdown of NCoR significantly reduced tumor volume and size compared with control (Fig. 4C). We next examined whether the inhibition of tumorigenecity is attributed to derepression of IP-10 and blocking the CK2-NCoR network. As shown in Fig. 4D, real-time PCR analyses using mrna from tumor samples demonstrate the level of IP-10 mrna is inversely associated with PC-3 cell *in vivo* tumorigenicity. This result suggests that IP-10 expression correlates with tumor growth but may not necessarily be causative. Collectively, these data indicate that the CK2-NCoR signaling network suppresses *in vivo* tumorigenecity of PC-3 cells.

### Inverse relationship between CK2-mediated NCoR phosphorylation and IP-10 expression during prostate cancer development

**Figure 5:**
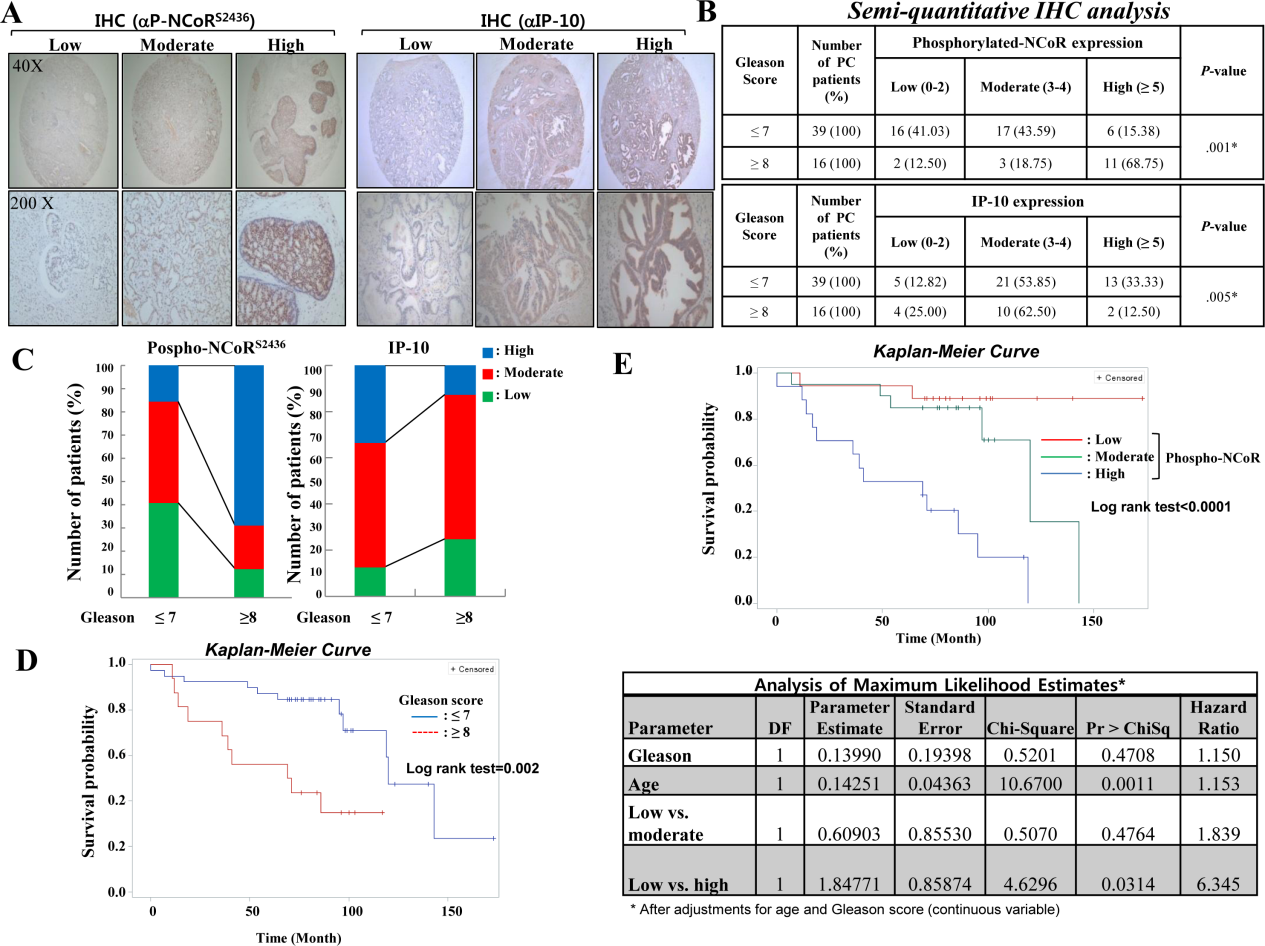
Inverse relationship between IP-10 and NCoR phosphorylation during prostate cancer development (A) Immunohistochemical staining of tissue sections was performed using phospho-NCoR and IP-10 antibodies at a 1:100 dilution. (B) Immunohistochemical staining of tissue sections was scored semi-quantitatively. The semi-quantitative analysis of immunohistochemical staining was calculated by: expression score = percentage of staining (0-3) + intensity score (0–3) among 55 patients with prostate cancer tissue. (C) Inverse relationship of phospho-NCoR with IP-10 expression between high-Gleason score (≥8) prostate cancer patients. (D) The survival rate of high-Gleason score (≥8) prostate cancer patients was significantly lower than that of low-Gleason score (≤7) prostate cancer patients according to Kaplan–Meier survival curve analyses. (E) Kaplan–Meier curves (upper panel) and Cox proportional hazard model analyses (lower panel) estimate the survival rate according to the phospho-NCoR level in the cohort. The Cox proportional hazard model analysis was performed after adjustments for age and Gleason score (continuous variable).

To further explore the clinical relevance of CK2-NCoR signaling and prostate cancer development, NCoR phosphorylation and IP-10 expression were evaluated by immunohistochemistry using tissue microarrays. Sixty two tissue samples from prostate cancer patients with diverse clinical stages were collected and semi-quantitative immunohistochemical staining using antibodies against NCoR Ser (P)-2436 and IP-10 was performed (Fig. 5A). From the original cohort of patients, 56 prostate carcinomas were available for analysis because of loss of some tissue cores during processing of the tissue microarrays. The intensity of immunohistochemical staining was indicated by an expression score, which combines the percentage of staining (0-3) with the intensity score (0-3) of 56 prostate cancer patients. Analysis of CK2-specific NCoR phosphorylation in prostate cancer patients revealed that 89% of samples (55 tissues) show positive staining for NCoR Ser (P)-2436, with 31% demonstrating a high score (≥5) for NCoR phosphorylation, 68.8% showing a high Gleason score (≥8), and 15.4% displaying a low Gleason score (≤7; Fig. 5B). High score of NCoR phosphorylation (≥5) was primarily observed in Gleason scores ≥8 (68.75%) than with Gleason scores ≤7 (15.38%), whereas the high score (≥5) of IP-10 was observed more often with Gleason scores ≤7 (33.3%) than with Gleason scores ≥8 (12.5%), providing the inverse relationship between NCoR phosphorylation and IP-10 in the late stage of prostate cancer development (Fig. 5C). As expected, the survival rate of high-Gleason score (≥8) patients was significantly lower than that of low-Gleason score (≤7) patients according to Kaplan–Meier survival curve analyses (Fig. 5D). Notably, univariate analysis indicated that expression of CK2-dependent NCoR phosphorylation significantly correlate with a decreased survival rate (Fig. 5E, upper panel). After stratification of the patients by Gleason score (≤7 and ≥8), the survival curves of patient with low Gleason score (≤7) were significantly different among NCoR phosphorylation status, but not in high Gleason score (≥8) group (Supplementary Figure 4). In the Cox proportional hazard model after adjustments for age and Gleason score (continuous variable), the risk of death was significantly increased in patients with high NCoR phosphorylation (hazard ratio, 6.35; p=0.03) compared with patients with low NCoR phosphorylation. evidence for the pathological relevance of CK2-dependent NCoR phosphorylation in prostate cancer development. Collectively, these results demonstrate that IP-10 levels inversely correlate with CK2-dependent NCoR phosphorylation levels during prostate cancer development.

## DISCUSSION

Discovering the molecular basis related to androgen-independent and hormone refractory prostate cancer progression is a central issue in the prostate tumor biology [32, 33]. Several molecular pathways, including AKK, MAPK, and NF-kB, have been suggested to explain hormone refractory prostate cancer survival and development [34]. In addition, the functional role of the AR coactivator and SRC/p160 protein complexes are well established and implicated in enhanced AR action after hormone deprivation therapy [35]. However, little is known about the roles of the corepressors NCoR and SMRT in androgen-independent prostate cancer development. In this study, we suggest a possible molecular mechanism by which NCoR modulates the invasive growth of androgen independent prostate cancer cells in a CK2-dependent manner. CK2 is known to participate in diverse cell signaling and aberrant expression of CK2 is believed to cause tumor development [36]. Thus, this kinase is considered a potential target for anti-cancer therapies. Here, we found constitutively elevated CK2 activity in androgen-independent DU145 and PC-3 cells, consistent with previous reports [19, 20]. We also found the pattern of NCoR phosphorylation and stability correlates with CK2 activity in prostate cancer cells. Our finding is consistent with a previous report that the NCoR1 protein is highly elevated in androgen-independent prostate cancer cell lines PC-3 and DU145 when compared with LNCaP and RWPE1 cells [29]. That study found NCoR1 to have diagnostic and prognostic significance in prostate tumor samples via antagonizing PPARα/γ signaling. On the other hand, we could not observe the significant increase of NCoR and NCoR phosphorylation in LNCaP-derivative C4-2B cells when compare to both DU145 and PC-3 cells, suggesting that NCoR phosphorylation by CK2 may be directly relevant to malignancy of prostate cancer cells rather that hormone independency. Further work should be performed to unravel this discrepancy among androgen-independent prostate cancer cells.

In a previous study, we determined that the CK2-NCoR cascade selectively represses the transcription of IP-10, which ultimately suppresses the invasion activity of esophageal cancer cells [18]. Here, we examined the relationship between IP-10 mRNA levels and CK2-mediated NCoR phosphorylation in PC-3 cells. Our results again displayed a reduced level of IP-10 in these cells with a concomitant elevation in CK2 activity similar to esophageal cancer cells. The varying levels of IP-10 among prostate cancer cell lines was explained by enhanced recruitment of the NCoR-HDAC3 corepressor complex to the AP-1 site of the IP-10 promoter in PC-3 cells compared with RWPE1 and LNCaP cells, which was reversed by blocking CK2 activity. In addition, inhibition of CK2 using TBB induced derepression of IP-10 and suppressed the invasive growth of PC-3 cells. Importantly, the transcriptional repression of IP-10 was selectively regulated by NCoR since knocking down NCoR had no effect on E-cadherin mRNA levels. Finally, ChIP analyses clearly demonstrated that the c-Jun-NCoR complex represses IP-10 transcription via histone deacetylation by HDAC3. Taken together, these results demonstrate the functional engagement of the NCoR-HDAC3 corepressor complex in regulating the invasion activity of PC-3 cells in a CK2-dependent manner.

Activation of EGFR signaling is also known to inhibit IP-10-mediated tumor cell migration [37, 38]. In addition, it has been recently shown that EGFR signaling phosphorylates and enhances the activity of CK2, thereby promoting tumor cell invasion [39]. Intriguingly, elevated expression of EGFR was recently reported to promote the tumorigenic growth of PC-3 cells [40]. Thus, it is possible that EGFR signaling enhances CK2-dependent NCoR phosphorylation and subsequently leads to the downregulation of IP-10 expression. Consistent with this hypothesis, we found that blocking EGFR signaling in PC-3 cells by ceteuximab treatment efficiently reversed the transcriptional repression of IP-10 as well as the stability and phosphorylation of NCoR (data not shown). However, we failed to observe the additional effect of EGF treatment on the level of IP-10 mRNA, which is likely due to the constitutive repression of IP-10 transcription in PC-3 cells. Therefore, CK2-mediated tumorigenic pathways may be controlled by extracellular receptor signaling; however, future studies are needed to address this issue.

Tumor xenograft assays in mice confirmed that inhibition of the CK2-NCoR signaling network suppresses the *in vivo* tumorigenecity of PC-3 cells with derepression of IP-10 expression observed. Furthermore, clinicopathological analyses clearly showed the increase of CK2-dependent NCoR phosphorylation is inversely associated with the survival probability of prostate cancer patients, confirming our hypothesis that NCoR promotes oncogenesis of prostate cancer in a CK2-dependent manner. In our study, we did not find an effect of NCoR phosphorylation on survival probability in patients with high Gleason scores. Among 16 patients with Gleason scores ≥8, however, most had high NCoR phosphorylation, and only two and three, respectively, had with low and moderate NCoR phosphorylation. Thus, due to the limitations of the small sample size in each NCoR phosphorylation group, we did not find a significant effect of NCoR phosphorylation on survival probability in patients with high NCoR phosphorylation. Further investigation with larger sample sizes will be necessary to confirm our results.

In summary, this study demonstrates that CK2 promotes tumorigenic growth of androgen-independent prostate cancer cells via NCoR-dependent repression of IP-10 pathway. Therefore, our study provide the rationale for androgen-independent prostate cancer therapy by disruption of CK2-NCoR signaling network.

## MATERIALS AND METHODS

### Cell Culture, plasmids, and antibodies

Both human PC-3 and LNCaP were from the American type culture collection (ATCC) (Rockville, Maryland, USA) and cultured in RPMI 1640 media (Invitrogen, Carlsbad, CA, USA) supplemented with 10% FBS. The CK2 inhibitor 4,5,6,7-tetrabromobenzotriazole (TBB) was prepared as a 50 mM stock solution in DMSO (Sigma-Aldrich, St. Louis, MO, USA). The RWPE1 cells were grown in keratinocyte serum-free medium (K-SFM) containing 50 μg/ml bovine pituitary extract and 5 ng/ml epidermal growth factor. The Control cultures received the same amount of DMSO as experimental cultures and final DMSO concentrations did not exceed 0.1%. Transient transfections were performed using Polyexpress (Excellgen, Rockville, MD, USA). The following antibodies were used: anti-CK2 (Millipore, Billerica, MA, USA), anti-HA (Sigma-Aldrich and Covance, Princeton, NJ, USA), anti-FLAG (Sigma-Aldrich), anti-β-actin (Sigma-Aldrich), anti-GAPDH (Millipore), anti-NCoR (ATGEN, Seongnam, Gyeonggido, Korea), anti-p300 (Millipore), anti-c-Jun (Epitomics, Burlingame, CA, USA), anti-c-Fos (Epitomics), anti-acetyl-histone H3 (Santa Cruz Biotechnology, Santa Cruz, CA, USA), anti-IP-10 (Santa Cruz Biotechnology), anti-HDAC3 (Santa Cruz Biotechnology), anti-SMRT (Millipore), and anti-Myc (Cell Signaling, Danvers, MA, USA). The phospho-NCoR antibody was described previously [18]. The CK2α construct was generated by PCR and cloned into the pSG5-KF2M1 and pSG5-KM2M1 (Sigma-Aldrich) plasmid vectors. Full-length pCMV-GFP-NCoR (1-2453) was previously described [41].

### siRNA and lentiviral shRNAs

The siRNAs against NCoR and CK2α as well as a non-specific siRNA were obtained from GenePharma (Shanghai, China). For siRNA transfection, PC-3 cells were incubated in serum and antibiotic free RPMI 1640 for 12 h and 200 nM nonspecific siRNA, siRNA-NCoR, and siRNA-CK2 were transfected using Lipofectamine 2000 following the manufacturer's protocol (Invitrogen). After 4 h, the media was changed and cells were incubated for 2 days. siRNAs against NCoR and CK2α were designed as follows: siNCoR forward (F): 5'-GGUGAUAAUACCAAAGAAATT-3'; reverse (R): 5'-UUUCUUUGGUAUUAUCACCTT-3' and siCK2α (F): 5'-CAGAAAGCUACGACUAAUATT-3'; (R): 5'-UAUUAGUCGUAGCUUUCUGTG-3'.

We established a stable PC-3 cell line with reduced NCoR expression using NCoR-specific shRNAs. To silence NCoR expression, two pairs of oligonucleotides coding for NCoR-specific shRNA were purchased as MISSION shRNAs (Sigma-Aldrich). Next, we prepared lentiviral particles using pLKO.1-PURO NCoR shRNA with a three-plasmid co-transfection following instructions from Invitrogen. PC-3 cells were then transfected with lentivirus. After 2 day incubation, lentivirus from the culture media was collected and concentrated with Centricon-plus-20 filters (Millipore). Lentivirus PURO shRNA was generated as a control.

### Real-time PCR analyses

Total RNA was isolated using the RNA Easyspin kit according to the manufacturer's instructions (Intron, Korea). Total RNA from each sample was reverse transcribed with random primers using a StrataScript™ reverse transcriptase kit (Stratagene), according to the manufacturer's protocol. RT–PCR analysis and quantification were performed with SYBR Green PCR Master Mix reagents (Applied Biosystems, Foster City, CA, USA) on an ABI Prism 7300 Sequence Detection System (Applied Biosystems). The specificity of the amplifications were verified using the Dissociation Analysis Software. All samples were normalized to human GAPDH. The primer sequences used for amplification of IP-10 were (F) 5'-CTGCCATTCTGATTTGCTGC-3' and (R) 5'-GATGGCCTTCGATTCTGGAT-3'. Primer sequences for amplification of E-cadherin RNA were 5'-AACGCATTGCCACATACATACACT-3' (F) and 5'-CCATGACAGACCCCTTAAAGA-3' (R). All reactions were performed in triplicate. Relative expression levels and standard deviations were calculated using the comparative method.

### In vitro kinase assay

GST-fusion proteins were incubated with 500 U of recombinant CK2α (ATGEN) in the presence of kinase reaction buffer (10 μl 5× kinase buffer, 10 μl magnesium/ATP cocktail solution 90 μl 75 mM MgCl_2_/500 mM ATP plus 10 μl [100 μCi] of [γ-^32^P]-ATP [3000 Ci/mmole]) in a total volume of 50 μl for 30 min at 30°C. Reactions were terminated by washing twice with 1× kinase buffer. Samples were resuspended in 15 μl 5× SDS sample loading buffer and boiled for 5 min. After electrophoresis, SDS polyacrylamide gels were stained with Coomassie blue and dried, and the phosphorylated products were visualized by autoradiography or quantified by PhosphorImager analysis.

### Clinical specimens

All specimens derived from patients were collected and archived under protocols approved by the Institutional Review Board of Yonsei University College of Medicine (4-2012-0473). Informed consent was not required by the local ethics board as the study was considered an anonymous chart review. Patient data was analyzed anonymously. Archival prostate cancer tissues in paraffin blocks were retrieved from the Department of Pathology at Gangnam Severance Hospital, Yonsei University College of Medicine. Cores of 3-mm diameter paraffin embedded tissues were obtained and transferred to recipient blocks to make tissue microarray blocks using Quick-Ray^®^ (UNITMA, Seoul, Korea). [42]

### Immunohistochemical staining

Serial 4-μm sections were prepared from tissue microarray blocks derived from 55 patients with prostate cancer. The sections were used for hematoxylin and eosin (HE) staining and immunohistochemical evaluation of phospho-NCoR1 and IP-10 expression. Immunohistochemical staining was performed using EnVision™ kit (DAKO, Glostrup, Denmark) according to the manufacturer's recommended procedure. Phospho-NCoR1 and IP-10 antibodies were used at a dilution of 1:100. Two investigators independently reviewed the stained slides without knowledge of the clinical data. The intensity of immunohistochemical staining was evaluated as follows: expression score = percentage of staining (0; no staining, 1; <25 cells are positive, 2; 25-50%, 3; >50%) + intensity score (0; no staining, 1; faint, 2; moderate, 3; strong) [43]. The expression score is categorized as low (0-2), moderate (3-4) and high (≥5).

### Xenograft experiments

Cell suspensions (100 μl 1× PBS containing 1.5 × 10^6^ NCoR-depleted PC-3 cells or control PC-3 cells) were injected subcutaneously into the right flank of 5-week-old athymic BALB/c nu/nu mice. Each experimental group included six mice. Tumor size was monitored closely and measured every 3 days using a caliper. Three weeks after injection, mice with comparable-sized tumors were selected for treatment with TBB. After 3 weeks of TBB treatment, mice were sacrificed and tumors were harvested, photographed, and weighed. The volume of tumors was estimated according the formula: *Volume = ½* × *a* × *b2*, where (*a*) and (*b*) represented the largest and smallest diameters, respectively. At the termination of the study, tumors were harvested and weighed. Animal studies were performed after obtaining approval according to the guidelines of the Institutional Animal Care committee of the National Cancer Center Korea (NCC-08-034).

### Chromatin immunoprecipitation assays (ChIP)

ChIP assays were performed with the indicated antibodies as described previously [44], but without SDS in all buffers. Eluted DNA was amplified with specific primers using the SYBR green PCR master mix (Applied Biosystems). Primers used in PCR were as follows: P1 (forward (F): 5'-CCAGGCATTGTTTGAACTGC-3'; reverse (R): 5'-AGCAAAAGATGTCTTGCACAAA-3'). P2 (forward (F): 5'-GACTACCTCTCTCTAGAACT-3'; reverse (R): 5'-GATCTCAACACGTGGACAAA-3'). All reactions were normalized relative to input activity and are presented as the mean ± standard deviation (SD) from three independent experiments.

### Cell Invasion Assays

In vitro cell invasiveness was determined by the ability of cells to transmigrate through a layer of extracellular matrix in Biocoat Matrigel invasion chambers (SPL Lifescience, Pocheon, Gyeonggido, Korea). Post-transfected cells (48 h) were trypsinized and seeded at a density of 2.0 × 10^4^ per insert. After 24 h, non-invading cells were removed with cotton swabs. Invading cells were fixed with 100% methanol and stained with 1% crystal violat (Sigma-Aldrich) before enumeration under an inverted microscope. Data are expressed as the mean ± SD of at least three independent experiments.

### Duolink in situ proximity ligation assay (PLA) analysis

Duolink *in situ* PLA analysis was performed per the manufacturer's instructions (OLink Biosciences, Uppsala, Sweden). In short, paraformaldehyde-fixed cells were washed with PBS, incubated for 15 min in 1.5% hydrogen peroxide, washed, and blocked with blocking solution. Primary rabbit antibody was applied and the cells were incubated with PLUS and MINUS secondary PLA probes against rabbit IgG only or against both rabbit and mouse IgG. The incubation was followed by hybridization, ligation, and amplification. After mounting with Duolink mounting medium, the samples were examined using an Olympus FluoView FV1000 Confocal Microscope (Olympus Corp., Tokyo, Japan).

### Statistical Analyses

Statistical analyses were performed using Student's *t*-tests with Bonferroni correction for multiple comparisons. A *p*-value less than 0.05 was considered statistically significant. Survival probability was measured from the time of surgery to disease progression or death, and analyzed using Kaplan-Meier method. The log-rank test was employed to compare the NCoR^S2436^ phosphorylation status and test the significance of Gleason score. Multivariate analysis was performed using the Cox proportional hazards model.

## SUPPLEMENTARY FIGURES


