# Supplementary material for: CK2-NCoR signaling cascade promotes prostate tumorigenesis
Source: Oncotarget. 2013 May 11;4(7):972–83. doi: 10.18632/oncotarget.1020 (PMC3759675; doi:10.18632/oncotarget.1020)
Supplement: Supplementary file 1 [file oncotarget-04-972-s001.pdf]

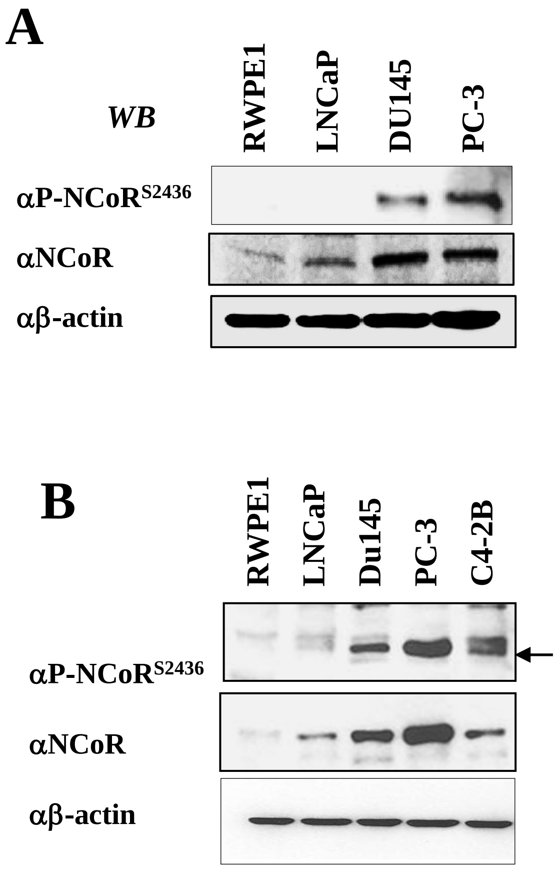

Figure S1: Constitutive elevation of NCoR^{S2436} phosphorylation in DU145 and PC-3 cells , LNCaP-derivative C4-2B cell line. Whole cell lysates were analyzed by western blotting with indicated antibodies.

Oncomine database analysis

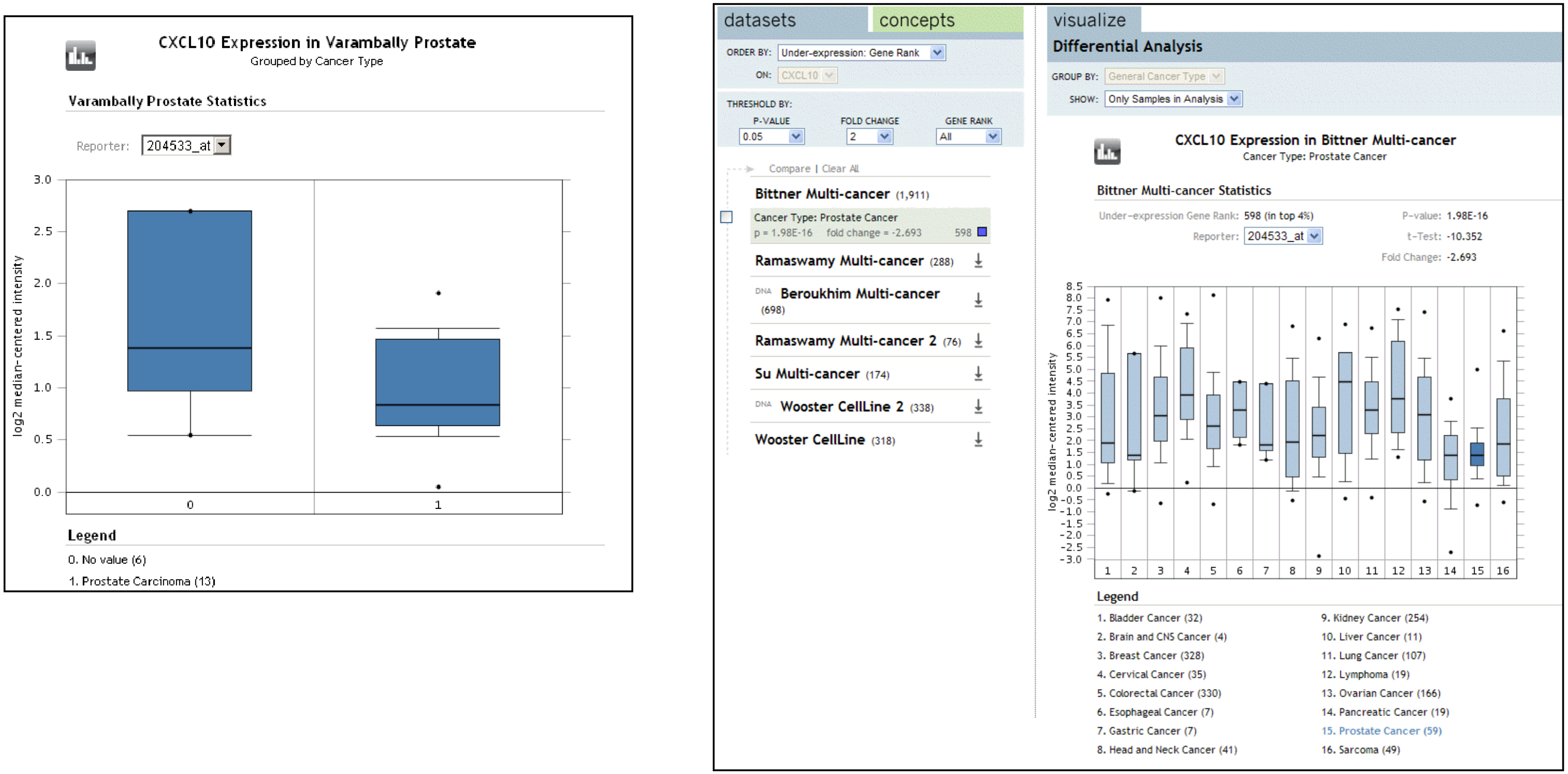

Figure S2: Decrease levels of IP-10 mRNA in prostate carcinoma. Oncomine 4.3 analysis for the expression patterns of IP-10 in normal and prostate cancer tissues.

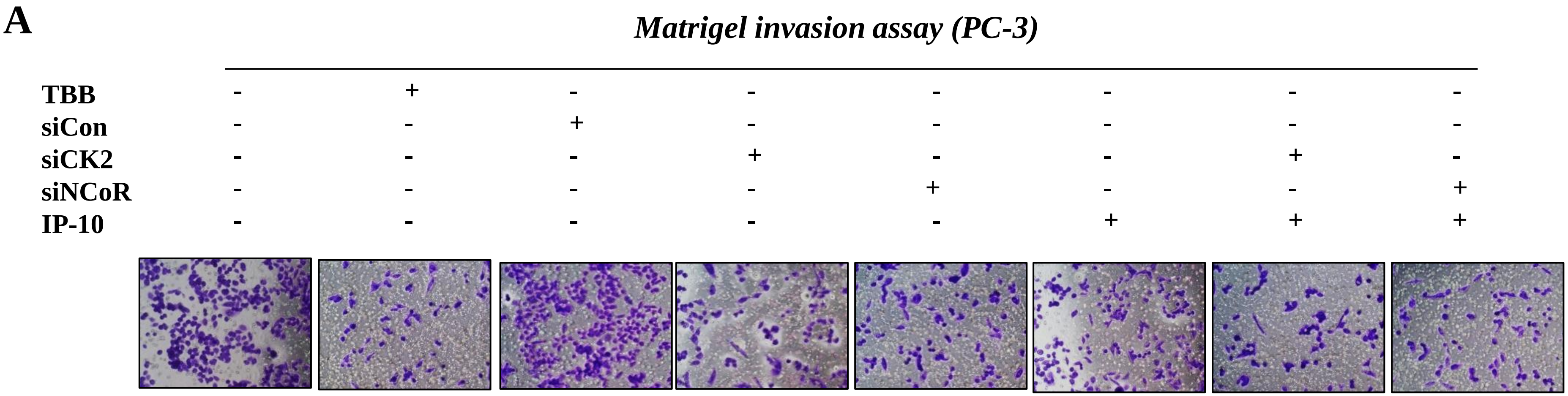

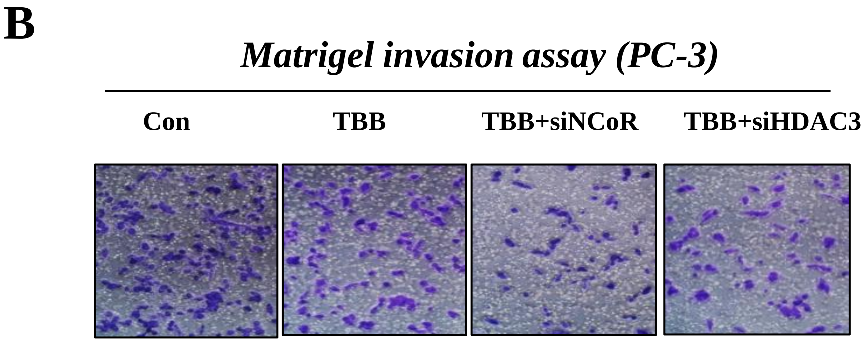

Figure S3: Blocking of CK2-NCoR axis suppresses the invasive growth of PC3 cells. PC3 cells were transfected with either siRNAs or IP-10 plasmid in the presence or absence of TBB. To measure the invasiveness of PC3 cells, the cells were transfected with the indicated plasmids and then seeded in the upper chamber. Cells that successfully invaded were stained with 1% crystal violat. Original magnification, ×400.

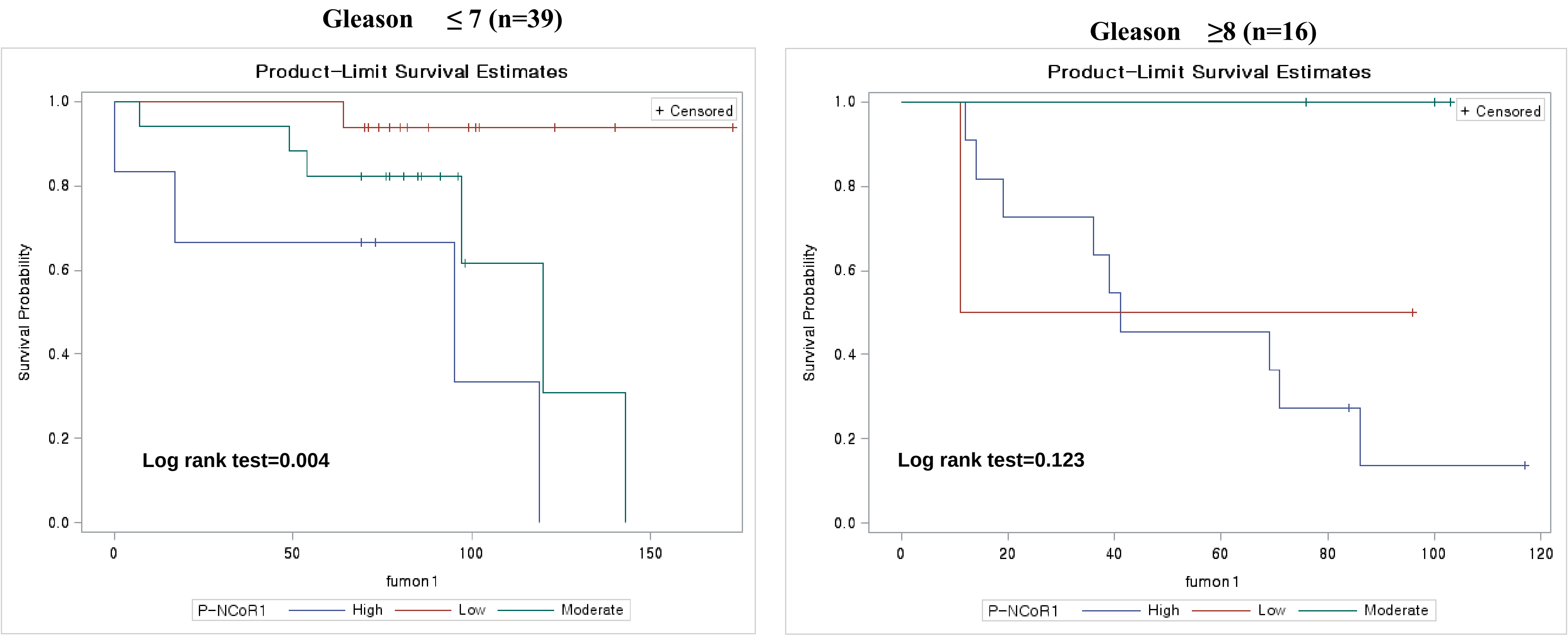

Figure S4: The survival curves of patients with low Gleason scores (≤7) were significantly different among NCoR phosphorylation statuses. We calculated the survival rates by Kaplan–Meier survival curve analyses after stratification of the patients by Gleason scores (≤7 and ≥8).
